# The Development of a Multicomponent Group Music Intervention for Persons with Dementia Living in Residential Care

**DOI:** 10.1093/geroni/igaf122.4249

**Published:** 2025-12-31

**Authors:** Silvia Orsulic-Jeras, Gregg Gorzelle, Lacey DiFranco, Michael Skrajner, Sara Powers, Sarah Nicolay, Emerson McSparran

**Affiliations:** Benjamin Rose, Cleveland, Ohio, United States; Hopeful Aging, WInchester, Massachusetts, United States; Benjamin Rose, Cleveland, Ohio, United States; Hopeful Aging, Winchester, Massachusetts, United States; Benjamin Rose, Cleveland, Ohio, United States; Benjamin Rose, Cleveland, Ohio, United States; Miami University, Oxford, Ohio, United States

## Abstract

Engagement-focused, nonpharmacological interventions are essential for enhancing quality of life among persons with dementia (PWD) in long-term care (LTC). Making Connections Thru Music (MCTM), a group-based intervention designed to be delivered by trained older adult volunteers, is grounded in the Comprehensive Process of Group Engagement (Cohen-Mansfield et al., 2017). Two pilot studies demonstrated strong acceptability, feasibility, and high levels of engagement and positive affect, supporting further development. This presentation illustrates how the theoretical underpinnings of MCTM inform its expansion into a comprehensive, technology-supported program designed for scalability. The enhanced intervention consists of two novel components: (1) an online volunteer training course providing dementia education, communication strategies, and facilitation skills; and (2) a tablet-based software application delivering a structured, step-by-step session protocol. Intervention design explicitly considers external elements (e.g., noise, lighting), personal attributes (e.g., communication and cognitive strengths), and stimulus characteristics (e.g., music selection, utilization of props). Evidence-based engagement techniques (e.g., external aids, discussion prompts, tactile materials) are incorporated to increase reminiscence, affect, and social interaction. Implications of intervention design choices and training content for volunteer preparedness and resident outcomes will be discussed. Next steps include a randomized controlled trial (RCT) across 12 assisted living and nursing home communities in Ohio and Massachusetts. The RCT will evaluate feasibility, fidelity, and preliminary outcomes on resident engagement and affect. If successful, MCTM can be the first scalable, volunteer-delivered group music intervention available for PWD living in residential care settings.

